# Reducing Preventive-to-E/M Code Conversion Using a Diagnosis-Triggered Documentation Template

**DOI:** 10.63116/AH.000000005

**Published:** 2026-09-01

**Authors:** Joseph E. Capito, Jonathon T. Williamson, Kelsey Samek, Kimberley Lanham, Erika Bodkins

**Affiliations:** 1 Department of Family Medicine, West Virginia University

**Keywords:** preventive health services, documentation, reimbursement mechanisms, primary health care, clinical coding

## Abstract

**Background:**

Preventive visits may be converted to problem-oriented evaluation and management (E/M) services when documentation does not clearly support preventive service criteria, resulting in lost reimbursement despite appropriate care delivery. The objective of this study was to evaluate whether a diagnosis-triggered documentation template embedded within the electronic health record reduces conversion of preventive visit codes to problem-oriented E/M codes.

**Methods:**

We conducted a 4-month observational evaluation in an academic family medicine clinic. A SmartText-based documentation template was triggered by the diagnosis of “annual physical examination” and inserted a standardized health care maintenance attestation into the clinical note. Encounters were classified as templated or nontemplated based on the presence of a SmartData Element. Preventive visit billing data were obtained from institutional reporting systems, and conversion rates were compared using a chi-square test, with effect size calculated using Cramér’s V.

**Results:**

Among 2310 preventive encounters, the template was used in 685 (29.7%). Overall, 37 visits (1.6%) were converted from preventive visit codes to problem-oriented E/M codes. Conversion occurred in 4 templated visits (0.6%) compared with 33 nontemplated visits (2.0%). Template use was associated with a statistically significant reduction in conversion (χ^2^ = 6.40, *df* = 1, *P* = .011), with a small effect size (Cramér’s V = 0.053).

**Conclusions:**

A diagnosis-triggered documentation template was associated with reduced conversion of preventive visit codes to problem-oriented E/M services. Relatively simple electronic health record (EHR) configuration changes may improve documentation consistency and influence downstream coding outcomes in primary care.

## Background

Preventive visits are a routine part of family medicine practice, but ensuring that documentation consistently meets preventive billing requirements across a practice can be challenging. Commercial payer preventive visit codes (Current Procedural Terminology [CPT] 99385-99387 and 99395-99397) generally require documentation reflecting a comprehensive preventive evaluation, including a review of medical, surgical, family, and social histories; performance of an age-appropriate physical examination; and discussion of preventive health recommendations. Preventive medicine services require documentation of an age-appropriate comprehensive evaluation and counseling. When documentation instead reflects evaluation or management of specific problems, coders may appropriately convert preventive visit codes to problem-oriented evaluation and management (E/M) services. Incomplete documentation can also lead to lost reimbursement when services provided are not adequately reflected in the medical record.^1^ Preventive services are intended to improve population health and reduce barriers to care, yet patients may still experience unexpected out-of-pocket costs when preventive visits are not coded as intended.^2^ Although documentation expectations vary by payer and audit process, clearly recording the preventive nature of the visit and the services provided supports appropriate coding.

Clinical documentation has traditionally served to record a patient’s clinical course and communicate clinical reasoning among members of the care team. However, modern documentation practices have increasingly been shaped by administrative and reimbursement requirements. The American College of Physicians has emphasized that documentation should primarily support patient care and communication while minimizing unnecessary administrative burden.^3^ At the same time, variation in how clinicians interact with the electronic health record (EHR) system can influence clinical workflows and operational outcomes, highlighting the importance of designing EHR tools that support consistent and efficient documentation.^4^

Our academic family medicine clinic includes faculty physicians, residents, fellows, and physician assistants, with new trainees joining the practice each year. Documentation practices varied across providers, and conversion from preventive visit codes to problem-oriented E/M codes occasionally occurred when preventive visit documentation did not clearly reflect the required elements.

To address this issue, we introduced a standardized annual examination documentation template within the EHR. The template was created in full by a physician in the practice with analyst privileges and appears as a quick-action button, available to all providers. When the diagnosis code for an annual examination is selected, the template automatically inserts a health care maintenance attestation summarizing the review of medical histories, physical examination findings, and preventive counseling. The goal of this tool was to standardize the workflow and documentation for preventive visits and reduce billing conversion.

This article describes the implementation of this template and evaluates its early impact during the first 4 months of formal tracking. We examined how often the template was used and whether its use was associated with lower rates of preventive visit billing conversion to E/M codes.

## Methods

The academic family medicine clinic in this study serves more than 20,000 patients and includes 23 faculty physicians, 18 residents, 2 fellows, and 4 physician assistants. Providers document visits using the Epic EHR.

To support consistent documentation for preventive visits and assist new providers and residents as they begin documenting in our clinic, we implemented standardized note templates within the ambulatory workflow. The templates appear as optional quick-action buttons in both the Progress Notes and History and Physical (H&P) sections of the Epic ambulatory documentation interface. Providers were educated on how to access the template and use its features but not required to use it in place of their existing workflow. Education consisted of a 1-time 5-minute demonstration at a department meeting.

These templates contain embedded SmartText that uses rule logic (in Epic, CERMSGREFRESH) to insert additional documentation when appropriate. Specifically, a patient rule evaluates the diagnoses assigned during the current visit and returns true if the visit includes the diagnosis of annual physical examination. Providers have been educated to use this diagnosis when documenting annual examination encounters, and it is available as a default quick-action option within the visit diagnosis section of the EHR.

When the “annual physical examination” diagnosis is present, the rule inserts a SmartText element that generates the following health care maintenance attestation in the assessment section of the note:

Healthcare Maintenance

@HMPENDING@ (lists health maintenance topics that are due, pended during the visit, or previously completed)

Past medical history, past surgical history, family history, social history reviewed. Physical examination within normal limit unless otherwise noted. Healthcare maintenance recommendations for preserving well-being were discussed.

This SmartText is embedded with a SmartData Element (SDE) labeled NORMAL ROUTINE HISTORY AND PHYSICAL with a value of “Yes.” Using Epic Report Workbench, a report can be generated for any specified time period to identify encounters in which this SDE was recorded. These encounters represent visits in which the preventive documentation attestation was inserted. The resulting list was cross referenced with monthly clinic billing reports of annual visits that were converted from an annual physical examination to another CPT code to calculate the rate of conversion.

We conducted a 4-month evaluation of preventive visit documentation beginning when formal tracking of the SDE began. Encounters in which the SDE was present were classified as templated visits.

Preventive visit billing data were obtained from the institutional Tableau reporting system using the professional billing (PB) CPT Dashboard. Encounters were filtered to include visits within the family medicine division with CPT codes for adult preventive visits (99385, 99386, 99387, 99395, and 99396) and service dates between November 1, 2025, and February 28, 2026. To identify encounters in which preventive visit codes were modified during coding review, a second Tableau report (PB Provider Coder Change Report) was used to identify visits in which the original preventive CPT code was changed by coders. Encounters in which the SDE was not present were classified as nontemplated visits.

Rates of billing conversion were calculated for templated and nontemplated visits. The association between template use and conversion was evaluated using a chi-square test, with statistical significance defined as *P* < .05. The effect size was calculated using Cramér’s V. This study was reviewed by the authors’ institutional review board and determined to be exempt.

## Results

During the 4-month evaluation period, 2310 preventive encounters were identified. The documentation template was used in 685 visits (29.7%), whereas 1625 visits (70.3%) were documented without the template. Overall, 37 visits (1.6%) were converted from preventive visit codes to problem-oriented E/M codes. Conversion occurred in 4 templated visits (0.6%) compared with 33 nontemplated visits (2.0%; Table 1). Use of the documentation template was associated with a statistically significant reduction in billing conversion (χ^2^ = 6.40, *df* = 1, *P* = .011), although the overall effect size was small (Cramér’s V = 0.053).

**Table 1.** Comparison of Conversion Rates Between Encounters Documented With a Diagnosis-Triggered Documentation Template (Templated) and Those Documented Without the Template (Nontemplated)

**Group**	**Total (n)**	**Converted (n)**	**Conversion Rate (%)**
Templated	685	4	0.60
Nontemplated	1625	33	2.00

Conversion was defined as a change from a preventive visit CPT code (99385–99387, 99395–99396) to a problem-oriented evaluation and management (E/M) code during coding review.

## Discussion

In this 4-month evaluation, use of a standardized preventive visit documentation template was associated with a lower rate of billing conversion for annual examinations. Although the absolute difference in conversion rate was small, templated visits were less likely to be changed than visits documented without the template. These findings suggest that relatively simple EHR configuration changes can influence documentation behavior and downstream coding outcomes without requiring changes to billing workflows or enforcement mechanisms. Even modest reductions in conversion can be meaningful for large primary care practices where preventive visits represent a substantial proportion of encounter volume.

Clinical documentation serves to communicate a patient’s clinical condition and the clinician’s reasoning to other members of the care team.^3^ Documentation requirements associated with reimbursement and regulatory oversight have increased the complexity of clinical notes, and variation in how clinicians interact with the EHR systems can influence documentation patterns and operational outcomes.^4^ The diagnosis-triggered template described in this study represents 1 example of how relatively simple EHR configuration changes may help standardize documentation across a diverse group of clinicians. Standardized documentation tools may help align clinical documentation with billing requirements while reducing the need for retrospective billing corrections.

Several limitations should be considered when interpreting these findings. First, template use was optional, and providers who chose to use the template may differ from those who did not. Differences in documentation habits or familiarity with preventive visit coding could influence conversion rates independently of the template itself. Second, the evaluation period was limited to 4 months, which may not fully capture long-term adoption patterns or sustained effects on billing outcomes. Third, template-based documentation can introduce risks if prepopulated text replaces individualized clinical documentation. Prior studies have described how copy-and-paste behaviors in EHRs can propagate outdated or inaccurate information in clinical documentation.^5–7^ Of particular concern here, templated attestations may create false reassurance that preventive elements were addressed when they were not, and diagnosis-triggered tools depend on accurate visit coding by the clinician. In this implementation, the template was intentionally limited to brief, structured language prompting documentation of preventive services and did not include prefabricated subjective findings, objective examination elements, or assessment and plan content. Clinicians remained responsible for documenting the actual preventive services performed during the encounter.

Despite these limitations, the findings suggest that relatively simple documentation tools may improve consistency in preventive visit documentation and reduce billing conversion. Because the intervention relies on commonly available EHR features, such as diagnosis-based rules, SmartText, and discrete data elements, similar approaches could potentially be implemented in other primary care settings. Future work could evaluate longer term adoption of the template, assess provider perceptions of documentation workflow, and explore whether similar approaches improve documentation or billing accuracy for other visit types. The small effect size suggests that this intervention alone is unlikely to substantially change practice-level billing performance, but it may still offer value for new clinics or for clinicians who are establishing documentation workflows in a new EHR environment.

## Conclusions

Overall, a diagnosis-triggered documentation attestation embedded within an EHR template was associated with a reduction in preventive visit billing conversion during early implementation. Structured documentation tools that guide clinicians toward including key preventive visit elements may represent a practical strategy for improving documentation consistency in busy primary care practices, as well as in new clinics or practices transitioning to a new EHR.

## Data Availability

The data that support the findings of this study are available from the corresponding author upon reasonable request.

## Conflicts Of Interest

The authors have nothing to disclose.

## Funding

The authors received no funding for this research.

CE Quiz

## References

[1] Jaqua EE, Chi R, Labib W, Uribe M, Najarro J, Hanna M. Optimize your documentation to improve Medicare reimbursement. Cleve Clin J Med. 2020;87(7):427-434. doi:10.3949/ccjm.87a.19116. 32605978

[2] Tran A, Laporte A, Nauenberg E, Hoagland A. Role of chronic conditions in out-of-pocket costs for preventive care in the US. JAMA Netw Open. 2026;9(1):e2553157. doi:10.1001/jamanetworkopen.2025.53157. PMC12784232 41505129

[3] Kuhn T, Basch P, Barr M, Yackel T. **Medical Informatics Committee of the American College of Physicians.** Clinical documentation in the 21st century: executive summary of a policy position paper from the American College of Physicians. Ann Intern Med. 2015;162(4):301-303. doi:10.7326/M14-2128. 25581028

[4] Baxter SL, Apathy NC, Cross DA, Sinsky C, Hribar MR. Measures of electronic health record use in outpatient settings across vendors. J Am Med Inform Assoc. 2021;28(5):955-959. doi:10.1093/jamia/ocaa266. PMC8068413 33211862

[5] Weir CR, Hicken BL, Nebeker JR, Campo R, Drews FA, LeBar B. Direct text entry in electronic progress notes: an evaluation of input errors. J Am Med Inform Assoc. 2010;17(3):282-286. doi:10.1136/jamia.2009.000448.

[6] Hirschtick RE. Copy-and-Paste. JAMA. 2006;295(20):2335–2336. doi:10.1001/jama.295.20.2335. 16720812

[7] Tsou AY, Lehmann CU, Michel J, Solomon R, Possanza L, Gandhi T. Safe Practices for Copy and Paste in the EHR. Systematic Review, Recommendations, and Novel Model for Health IT Collaboration. Appl Clin Inform. 2017;8(1):12–34. doi:10.4338/ACI-2016-09-R-0150. PMC5373750 28074211

